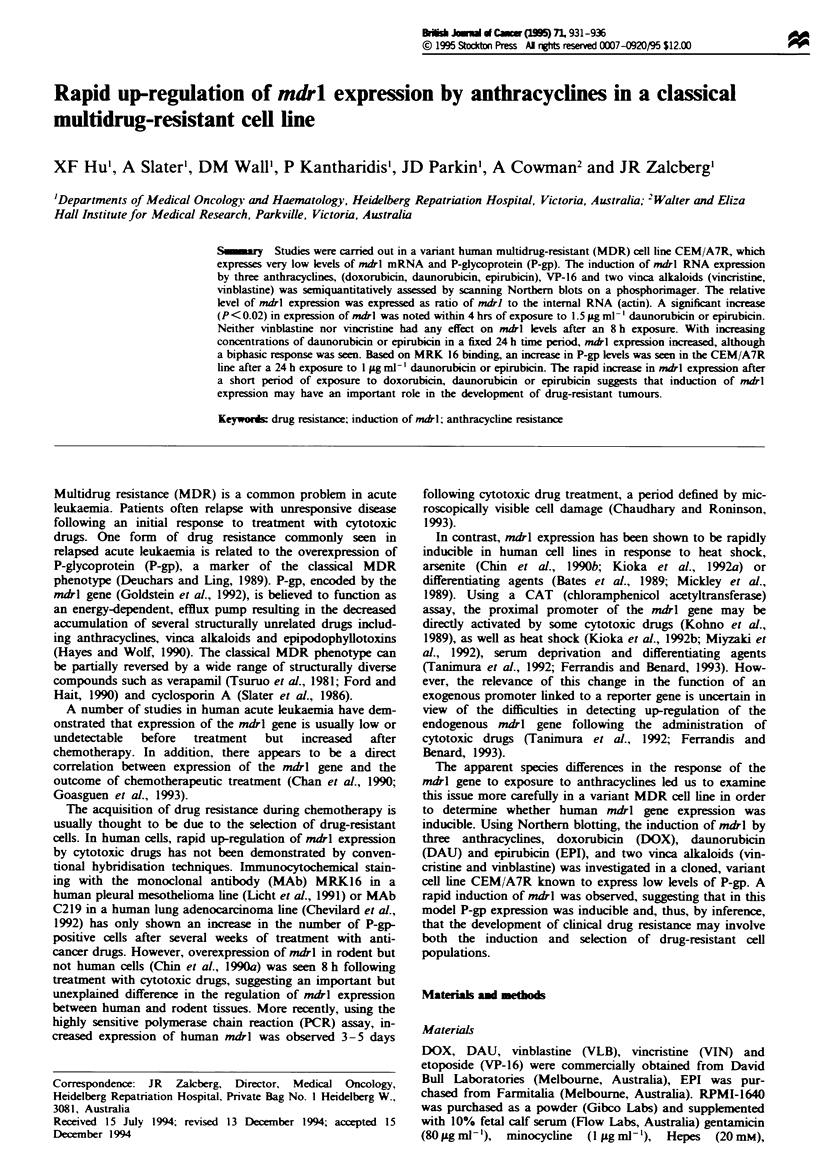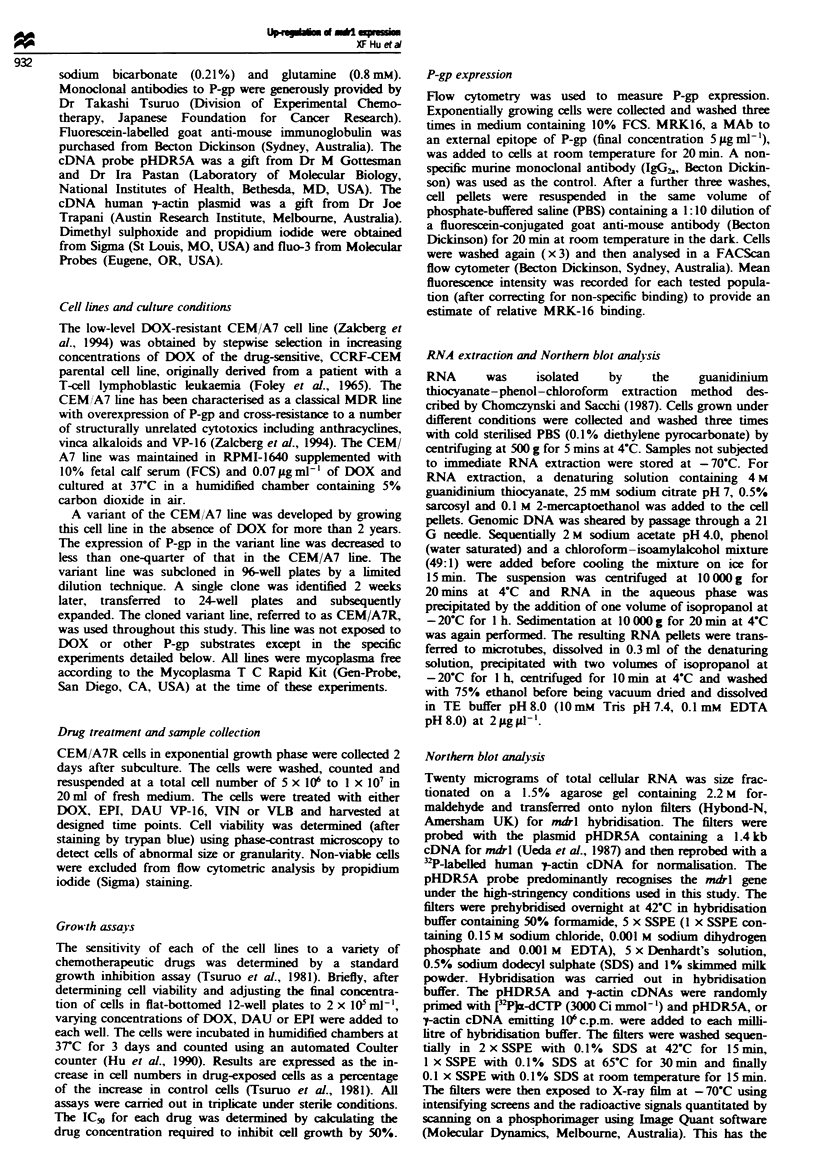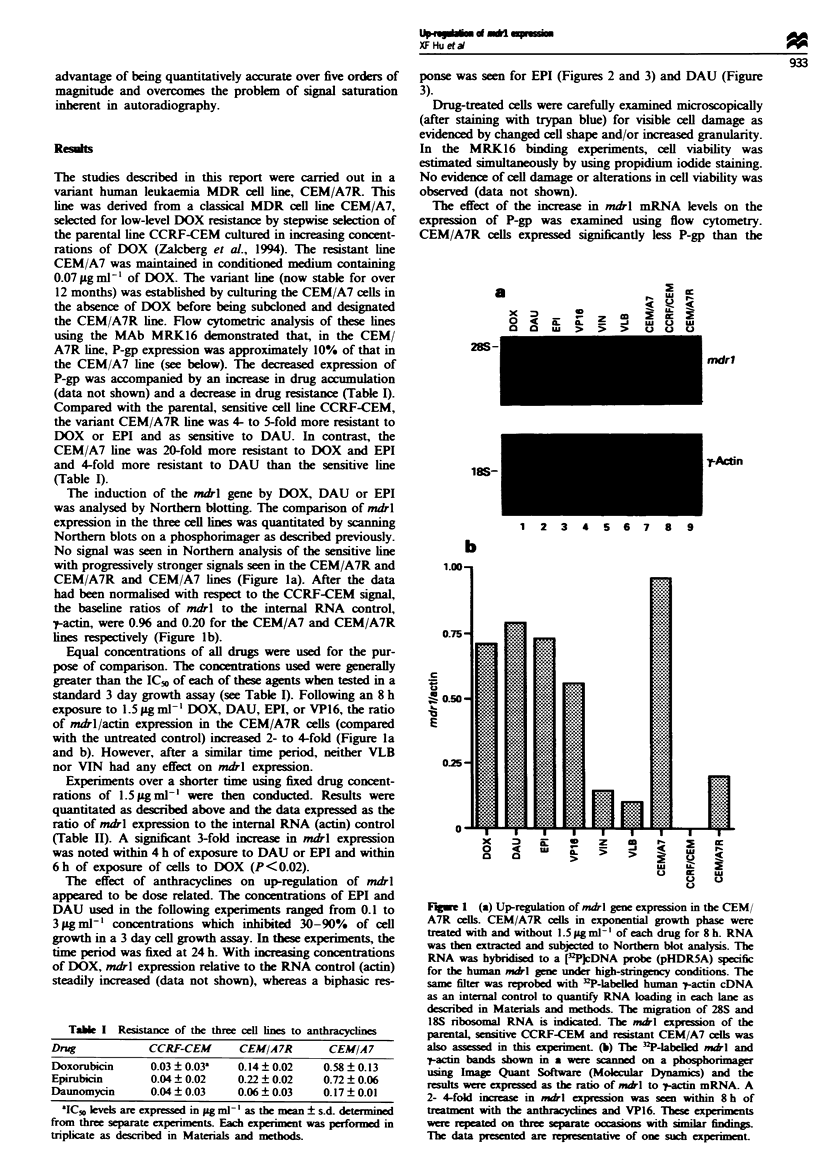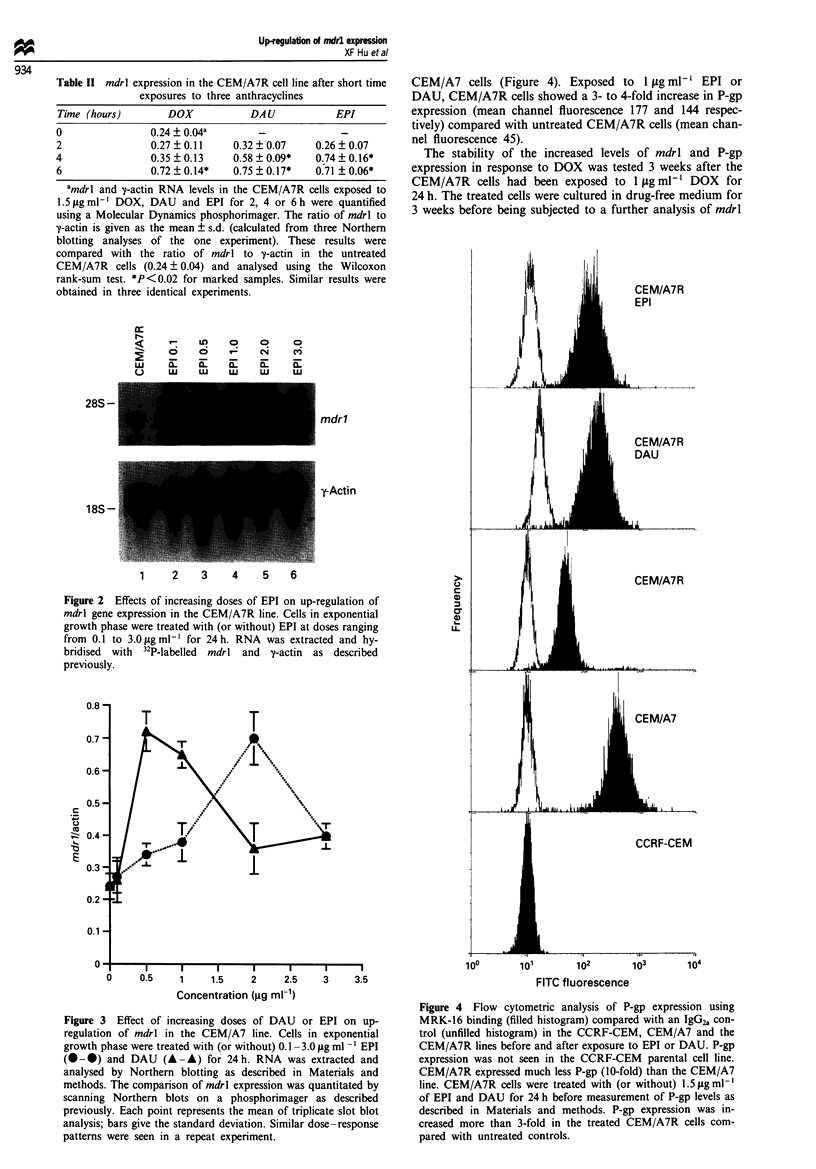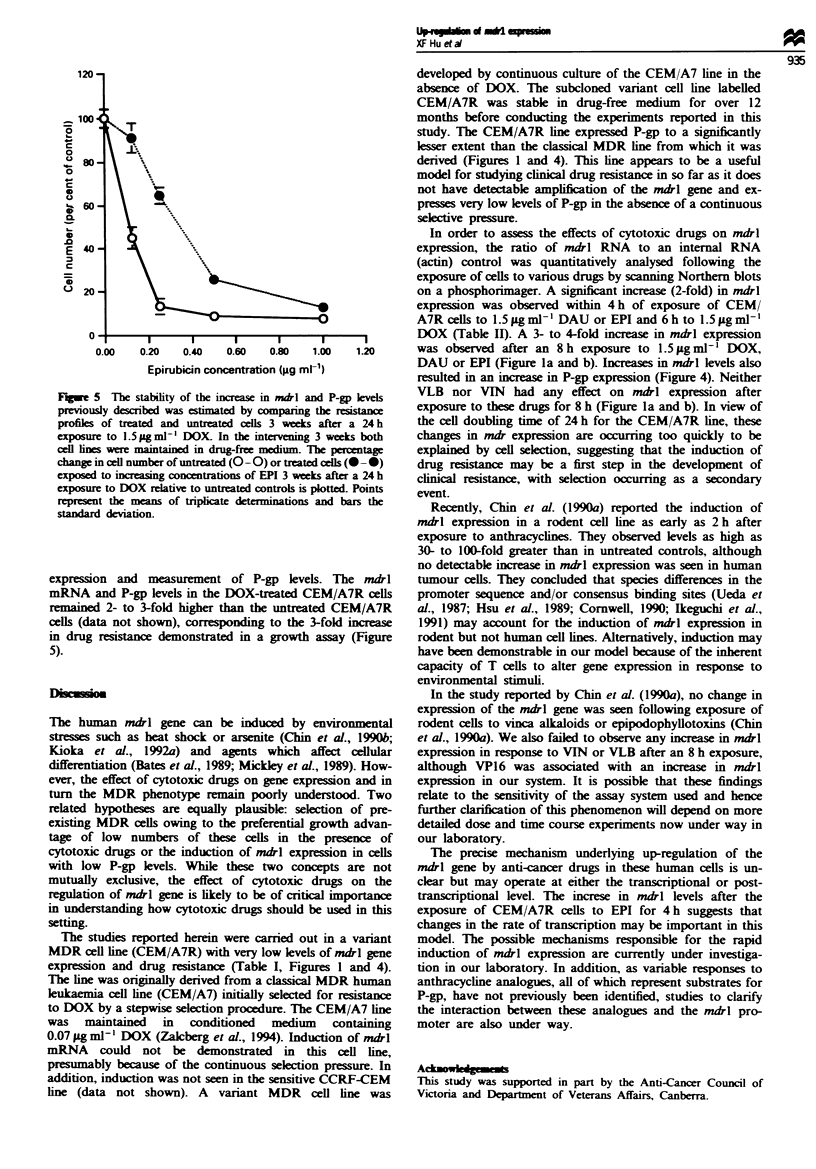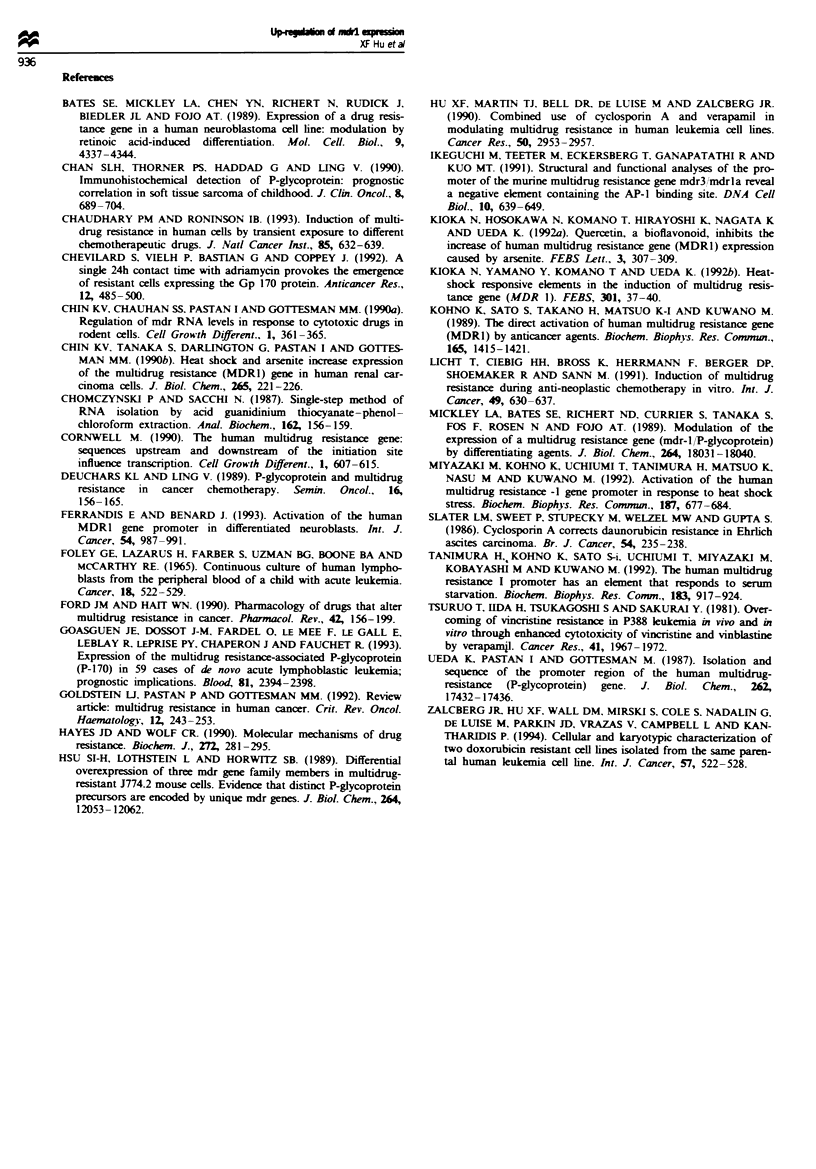# Rapid up-regulation of mdr1 expression by anthracyclines in a classical multidrug-resistant cell line.

**DOI:** 10.1038/bjc.1995.180

**Published:** 1995-05

**Authors:** X. F. Hu, A. Slater, D. M. Wall, P. Kantharidis, J. D. Parkin, A. Cowman, J. R. Zalcberg

**Affiliations:** Department of Medical Oncology, Heidelberg Repatriation Hospital, Victoria, Australia.

## Abstract

**Images:**


© 1995 Stockton Press All rights reserved 0007-0920/95 $12.00

### Rapid up-regulation of *mdr*1 expression by anthracyclines in a classical multidrug-resistant cell line

XF Hu[1], A Slater[1], DM Wall[1], P Kantharidis[1], JD Parkin[1], A Cowman[2] and JR Zalcberg[1]

[1]*Departments of Medical Oncology and Haematology, Heidelberg Repatriation Hospital, Victoria, Australia;* [2]*Walter and Eliza Hall Institute for Medical Research, Parkville, Victoria, Australia*

**Summary** Studies were carried out in a variant human multidrug-resistant (MDR) cell line CEM/A7R, which expresses very low levels of *mdr*1 mRNA and P-glycoprotein (P-gp). The induction of *mdr*1 RNA expression by three anthracyclines, (doxorubicin, daunorubicin, epirubicin), VP-16 and two vinca alkaloids (vincristine, vinblastine) was semiquantitatively assessed by scanning Northern blots on a phosphorimager. The relative level of *mdr*1 expression was expressed as ratio of *mdr1* to the internal RNA (actin). A significant increase ($P < 0.02$) in expression of *mdr*1 was noted within 4 hrs of exposure to 1.5 μg ml$^{-1}$ daunorubicin or epirubicin. Neither vinblastine nor vincristine had any effect on *mdr*1 levels after an 8 h exposure. With increasing concentrations of daunorubicin or epirubicin in a fixed 24 h time period, *mdr*1 expression increased, although a biphasic response was seen. Based on MRK 16 binding, an increase in P-gp levels was seen in the CEM/A7R line after a 24 h exposure to 1 μg ml$^{-1}$ daunorubicin or epirubicin. The rapid increase in *mdr*1 expression after a short period of exposure to doxorubicin, daunorubicin or epirubicin suggests that induction of *mdr*1 expression may have an important role in the development of drug-resistant tumours.

**Keywords:** drug resistance; induction of *mdr*1; anthracycline resistance

Multidrug resistance (MDR) is a common problem in acute leukaemia. Patients often relapse with unresponsive disease following an initial response to treatment with cytotoxic drugs. One form of drug resistance commonly seen in relapsed acute leukaemia is related to the overexpression of P-glycoprotein (P-gp), a marker of the classical MDR phenotype (Deuchars and Ling, 1989). P-gp, encoded by the *mdr*1 gene (Goldstein *et al.*, 1992), is believed to function as an energy-dependent, efflux pump resulting in the decreased accumulation of several structurally unrelated drugs including anthracyclines, vinca alkaloids and epipodophyllotoxins (Hayes and Wolf, 1990). The classical MDR phenotype can be partially reversed by a wide range of structurally diverse compounds such as verapamil (Tsuruo *et al.*, 1981; Ford and Hait, 1990) and cyclosporin A (Slater *et al.*, 1986).

A number of studies in human acute leukaemia have demonstrated that expression of the *mdr*1 gene is usually low or undetectable before treatment but increased after chemotherapy. In addition, there appears to be a direct correlation between expression of the *mdr*1 gene and the outcome of chemotherapeutic treatment (Chan *et al.*, 1990; Goasguen *et al.*, 1993).

The acquisition of drug resistance during chemotherapy is usually thought to be due to the selection of drug-resistant cells. In human cells, rapid up-regulation of *mdr*1 expression by cytotoxic drugs has not been demonstrated by conventional hybridisation techniques. Immunocytochemical staining with the monoclonal antibody (MAb) MRK16 in a human pleural mesothelioma line (Licht *et al.*, 1991) or MAb C219 in a human lung adenocarcinoma line (Chevilard *et al.*, 1992) has only shown an increase in the number of P-gp-positive cells after several weeks of treatment with anticancer drugs. However, overexpression of *mdr*1 in rodent but not human cells (Chin *et al.*, 1990*a*) was seen 8 h following treatment with cytotoxic drugs, suggesting an important but unexplained difference in the regulation of *mdr*1 expression between human and rodent tissues. More recently, using the highly sensitive polymerase chain reaction (PCR) assay, increased expression of human *mdr*1 was observed 3-5 days following cytotoxic drug treatment, a period defined by microscopically visible cell damage (Chaudhary and Roninson, 1993).

In contrast, *mdr*1 expression has been shown to be rapidly inducible in human cell lines in response to heat shock, arsenite (Chin *et al.*, 1990*b*; Kioka *et al.*, 1992*a*) or differentiating agents (Bates *et al.*, 1989; Mickley *et al.*, 1989). Using a CAT (chloramphenicol acetyltransferase) assay, the proximal promoter of the *mdr*1 gene may be directly activated by some cytotoxic drugs (Kohno *et al.*, 1989), as well as heat shock (Kioka *et al.*, 1992b; Miyzaki *et al.*, 1992), serum deprivation and differentiating agents (Tanimura *et al.*, 1992; Ferrandis and Benard, 1993). However, the relevance of this change in the function of an exogenous promoter linked to a reporter gene is uncertain in view of the difficulties in detecting up-regulation of the endogenous *mdr*1 gene following the administration of cytotoxic drugs (Tanimura *et al.*, 1992; Ferrandis and Benard, 1993).

The apparent species differences in the response of the *mdr*1 gene to exposure to anthracyclines led us to examine this issue more carefully in a variant MDR cell line in order to determine whether human *mdr*1 gene expression was inducible. Using Northern blotting, the induction of *mdr*1 by three anthracyclines, doxorubicin (DOX), daunorubicin (DAU) and epirubicin (EPI), and two vinca alkaloids (vincristine and vinblastine) was investigated in a cloned, variant cell line CEM/A7R known to express low levels of P-gp. A rapid induction of *mdr*1 was observed, suggesting that in this model P-gp expression was inducible and, thus, by inference, that the development of clinical drug resistance may involve both the induction and selection of drug-resistant cell populations.

### Materials and methods

### *Materials*

DOX, DAU, vinblastine (VLB), vincristine (VIN) and etoposide (VP-16) were commercially obtained from David Bull Laboratories (Melbourne, Australia), EPI was purchased from Farmitalia (Melbourne, Australia). RPMI-1640 was purchased as a powder (Gibco Labs) and supplemented with 10% fetal calf serum (Flow Labs, Australia) gentamicin (80 μg ml$^{-1}$), minocycline (1 μg ml$^{-1}$), Hepes (20 mM),

Correspondence: JR Zalcberg, Director, Medical Oncology, Heidelberg Repatriation Hospital, Private Bag No. 1 Heidelberg W., 3081, Australia
Received 15 July 1994; revised 13 December 1994; accepted 15 December 1994

sodium bicarbonate (0.21%) and glutamine (0.8 mM). Monoclonal antibodies to P-gp were generously provided by Dr Takashi Tsuruo (Division of Experimental Chemotherapy, Japanese Foundation for Cancer Research). Fluorescein-labelled goat anti-mouse immunoglobulin was purchased from Becton Dickinson (Sydney, Australia). The cDNA probe pHDR5A was a gift from Dr M Gottesman and Dr Ira Pastan (Laboratory of Molecular Biology, National Institutes of Health, Bethesda, MD, USA). The cDNA human γ-actin plasmid was a gift from Dr Joe Trapani (Austin Research Institute, Melbourne, Australia). Dimethyl sulphoxide and propidium iodide were obtained from Sigma (St Louis, MO, USA) and fluo-3 from Molecular Probes (Eugene, OR, USA).

*Cell lines and culture conditions*

The low-level DOX-resistant CEM/A7 cell line (Zalcberg *et al.*, 1994) was obtained by stepwise selection in increasing concentrations of DOX of the drug-sensitive, CCRF-CEM parental cell line, originally derived from a patient with a T-cell lymphoblastic leukaemia (Foley *et al.*, 1965). The CEM/A7 line has been characterised as a classical MDR line with overexpression of P-gp and cross-resistance to a number of structurally unrelated cytotoxics including anthracyclines, vinca alkaloids and VP-16 (Zalcberg *et al.*, 1994). The CEM/A7 line was maintained in RPMI-1640 supplemented with 10% fetal calf serum (FCS) and 0.07 $\mu g\ ml^{-1}$ of DOX and cultured at 37°C in a humidified chamber containing 5% carbon dioxide in air.

A variant of the CEM/A7 line was developed by growing this cell line in the absence of DOX for more than 2 years. The expression of P-gp in the variant line was decreased to less than one-quarter of that in the CEM/A7 line. The variant line was subcloned in 96-well plates by a limited dilution technique. A single clone was identified 2 weeks later, transferred to 24-well plates and subsequently expanded. The cloned variant line, referred to as CEM/A7R, was used throughout this study. This line was not exposed to DOX or other P-gp substrates except in the specific experiments detailed below. All lines were mycoplasma free according to the Mycoplasma T C Rapid Kit (Gen-Probe, San Diego, CA, USA) at the time of these experiments.

*Drug treatment and sample collection*

CEM/A7R cells in exponential growth phase were collected 2 days after subculture. The cells were washed, counted and resuspended at a total cell number of $5 \times 10^6$ to $1 \times 10^7$ in 20 ml of fresh medium. The cells were treated with either DOX, EPI, DAU VP-16, VIN or VLB and harvested at designed time points. Cell viability was determined (after staining by trypan blue) using phase-contrast microscopy to detect cells of abnormal size or granularity. Non-viable cells were excluded from flow cytometric analysis by propidium iodide (Sigma) staining.

*Growth assays*

The sensitivity of each of the cell lines to a variety of chemotherapeutic drugs was determined by a standard growth inhibition assay (Tsuruo *et al.*, 1981). Briefly, after determining cell viability and adjusting the final concentration of cells in flat-bottomed 12-well plates to $2 \times 10^5\ ml^{-1}$, varying concentrations of DOX, DAU or EPI were added to each well. The cells were incubated in humidified chambers at 37°C for 3 days and counted using an automated Coulter counter (Hu *et al.*, 1990). Results are expressed as the increase in cell numbers in drug-exposed cells as a percentage of the increase in control cells (Tsuruo *et al.*, 1981). All assays were carried out in triplicate under sterile conditions. The $IC_{50}$ for each drug was determined by calculating the drug concentration required to inhibit cell growth by 50%.

*P-gp expression*

Flow cytometry was used to measure P-gp expression. Exponentially growing cells were collected and washed three times in medium containing 10% FCS. MRK16, a MAb to an external epitope of P-gp (final concentration 5 $\mu g\ ml^{-1}$), was added to cells at room temperature for 20 min. A non-specific murine monoclonal antibody ($IgG_{2a}$, Becton Dickinson) was used as the control. After a further three washes, cell pellets were resuspended in the same volume of phosphate-buffered saline (PBS) containing a 1:10 dilution of a fluorescein-conjugated goat anti-mouse antibody (Becton Dickinson) for 20 min at room temperature in the dark. Cells were washed again (×3) and then analysed in a FACScan flow cytometer (Becton Dickinson, Sydney, Australia). Mean fluorescence intensity was recorded for each tested population (after correcting for non-specific binding) to provide an estimate of relative MRK-16 binding.

*RNA extraction and Northern blot analysis*

RNA was isolated by the guanidinium thiocyanate–phenol–chloroform extraction method described by Chomczynski and Sacchi (1987). Cells grown under different conditions were collected and washed three times with cold sterilised PBS (0.1% diethylene pyrocarbonate) by centrifuging at 500 g for 5 mins at 4°C. Samples not subjected to immediate RNA extraction were stored at −70°C. For RNA extraction, a denaturing solution containing 4 M guanidinium thiocyanate, 25 mM sodium citrate pH 7, 0.5% sarcosyl and 0.1 M 2-mercaptoethanol was added to the cell pellets. Genomic DNA was sheared by passage through a 21 G needle. Sequentially 2 M sodium acetate pH 4.0, phenol (water saturated) and a chloroform–isoamylalcohol mixture (49:1) were added before cooling the mixture on ice for 15 min. The suspension was centrifuged at 10 000 g for 20 mins at 4°C and RNA in the aqueous phase was precipitated by the addition of one volume of isopropanol at −20°C for 1 h. Sedimentation at 10 000 g for 20 min at 4°C was again performed. The resulting RNA pellets were transferred to microtubes, dissolved in 0.3 ml of the denaturing solution, precipitated with two volumes of isopropanol at −20°C for 1 h, centrifuged for 10 min at 4°C and washed with 75% ethanol before being vacuum dried and dissolved in TE buffer pH 8.0 (10 mM Tris pH 7.4, 0.1 mM EDTA pH 8.0) at 2 $\mu g\ \mu l^{-1}$.

*Northern blot analysis*

Twenty micrograms of total cellular RNA was size fractionated on a 1.5% agarose gel containing 2.2 M formaldehyde and transferred onto nylon filters (Hybond-N, Amersham UK) for *mdr*1 hybridisation. The filters were probed with the plasmid pHDR5A containing a 1.4 kb cDNA for *mdr*1 (Ueda *et al.*, 1987) and then reprobed with a $^{32}P$-labelled human γ-actin cDNA for normalisation. The pHDR5A probe predominantly recognises the *mdr*1 gene under the high-stringency conditions used in this study. The filters were prehybridised overnight at 42°C in hybridisation buffer containing 50% formamide, 5 × SSPE (1 × SSPE containing 0.15 M sodium chloride, 0.001 M sodium dihydrogen phosphate and 0.001 M EDTA), 5 × Denhardt's solution, 0.5% sodium dodecyl sulphate (SDS) and 1% skimmed milk powder. Hybridisation was carried out in hybridisation buffer. The pHDR5A and γ-actin cDNAs were randomly primed with [$^{32}P$]α-dCTP (3000 Ci $mmol^{-1}$) and pHDR5A, or γ-actin cDNA emitting $10^6$ c.p.m. were added to each millilitre of hybridisation buffer. The filters were washed sequentially in 2 × SSPE with 0.1% SDS at 42°C for 15 min, 1 × SSPE with 0.1% SDS at 65°C for 30 min and finally 0.1 × SSPE with 0.1% SDS at room temperature for 15 min. The filters were then exposed to X-ray film at −70°C using intensifying screens and the radioactive signals quantitated by scanning on a phosphorimager using Image Quant software (Molecular Dynamics, Melbourne, Australia). This has the advantage of being quantitatively accurate over five orders of magnitude and overcomes the problem of signal saturation inherent in autoradiography.

### Results

The studies described in this report were carried out in a variant human leukaemia MDR cell line, CEM/A7R. This line was derived from a classical MDR cell line CEM/A7, selected for low-level DOX resistance by stepwise selection of the parental line CCRF-CEM cultured in increasing concentrations of DOX (Zalcberg *et al.*, 1994). The resistant line CEM/A7 was maintained in conditioned medium containing 0.07 $\mu g\ ml^{-1}$ of DOX. The variant line (now stable for over 12 months) was established by culturing the CEM/A7 cells in the absence of DOX before being subcloned and designated the CEM/A7R line. Flow cytometric analysis of these lines using the MAb MRK16 demonstrated that, in the CEM/A7R line, P-gp expression was approximately 10% of that in the CEM/A7 line (see below). The decreased expression of P-gp was accompanied by an increase in drug accumulation (data not shown) and a decrease in drug resistance (Table I). Compared with the parental, sensitive cell line CCRF-CEM, the variant CEM/A7R line was 4- to 5-fold more resistant to DOX or EPI and as sensitive to DAU. In contrast, the CEM/A7 line was 20-fold more resistant to DOX and EPI and 4-fold more resistant to DAU than the sensitive line (Table I).

The induction of the *mdr*1 gene by DOX, DAU or EPI was analysed by Northern blotting. The comparison of *mdr*1 expression in the three cell lines was quantitated by scanning Northern blots on a phosphorimager as described previously. No signal was seen in Northern analysis of the sensitive line with progressively stronger signals seen in the CEM/A7R and CEM/A7R and CEM/A7 lines (Figure 1a). After the data had been normalised with respect to the CCRF-CEM signal, the baseline ratios of *mdr*1 to the internal RNA control, γ-actin, were 0.96 and 0.20 for the CEM/A7 and CEM/A7R lines respectively (Figure 1b).

Equal concentrations of all drugs were used for the purpose of comparison. The concentrations used were generally greater than the $IC_{50}$ of each of these agents when tested in a standard 3 day growth assay (see Table I). Following an 8 h exposure to 1.5 $\mu g\ ml^{-1}$ DOX, DAU, EPI, or VP16, the ratio of *mdr*1/actin expression in the CEM/A7R cells (compared with the untreated control) increased 2- to 4-fold (Figure 1a and b). However, after a similar time period, neither VLB nor VIN had any effect on *mdr*1 expression.

Experiments over a shorter time using fixed drug concentrations of 1.5 $\mu g\ ml^{-1}$ were then conducted. Results were quantitated as described above and the data expressed as the ratio of *mdr*1 expression to the internal RNA (actin) control (Table II). A significant 3-fold increase in *mdr*1 expression was noted within 4 h of exposure to DAU or EPI and within 6 h of exposure of cells to DOX ($P<0.02$).

The effect of anthracyclines on up-regulation of *mdr*1 appeared to be dose related. The concentrations of EPI and DAU used in the following experiments ranged from 0.1 to 3 $\mu g\ ml^{-1}$ concentrations which inhibited 30–90% of cell growth in a 3 day cell growth assay. In these experiments, the time period was fixed at 24 h. With increasing concentrations of DOX, *mdr*1 expression relative to the RNA control (actin) steadily increased (data not shown), whereas a biphasic response was seen for EPI (Figures 2 and 3) and DAU (Figure 3).

Drug-treated cells were carefully examined microscopically (after staining with trypan blue) for visible cell damage as evidenced by changed cell shape and/or increased granularity. In the MRK16 binding experiments, cell viability was estimated simultaneously by using propidium iodide staining. No evidence of cell damage or alterations in cell viability was observed (data not shown).

The effect of the increase in *mdr*1 mRNA levels on the expression of P-gp was examined using flow cytometry. CEM/A7R cells expressed significantly less P-gp than the

**Table I** Resistance of the three cell lines to anthracyclines

| *Drug* | *CCRF-CEM* | *CEM/A7R* | *CEM/A7* |
|---|---|---|---|
| Doxorubicin | 0.03 ± 0.03[a] | 0.14 ± 0.02 | 0.58 ± 0.13 |
| Epirubicin | 0.04 ± 0.02 | 0.22 ± 0.02 | 0.72 ± 0.06 |
| Daunomycin | 0.04 ± 0.03 | 0.06 ± 0.03 | 0.17 ± 0.01 |

[a]$IC_{50}$ levels are expressed in $\mu g\ ml^{-1}$ as the mean ± s.d. determined from three separate experiments. Each experiment was performed in triplicate as described in Materials and methods.

**Figure 1** (**a**) Up-regulation of *mdr*1 gene expression in the CEM/A7R cells. CEM/A7R cells in exponential growth phase were treated with and without 1.5 $\mu g\ ml^{-1}$ of each drug for 8 h. RNA was then extracted and subjected to Northern blot analysis. The RNA was hybridised to a [$^{32}$P]cDNA probe (pHDR5A) specific for the human *mdr*1 gene under high-stringency conditions. The same filter was reprobed with $^{32}$P-labelled human γ-actin cDNA as an internal control to quantify RNA loading in each lane as described in Materials and methods. The migration of 28S and 18S ribosomal RNA is indicated. The *mdr*1 expression of the parental, sensitive CCRF-CEM and resistant CEM/A7 cells was also assessed in this experiment. (**b**) The $^{32}$P-labelled *mdr*1 and γ-actin bands shown in **a** were scanned on a phosphorimager using Image Quant Software (Molecular Dynamics) and the results were expressed as the ratio of *mdr*1 to γ-actin mRNA. A 2- 4-fold increase in *mdr*1 expression was seen within 8 h of treatment with the anthracyclines and VP16. These experiments were repeated on three separate occasions with similar findings. The data presented are representative of one such experiment.

**Table II** *mdr*1 expression in the CEM/A7R cell line after short time exposures to three anthracyclines

| *Time (hours)* | *DOX* | *DAU* | *EPI* |
|---|---|---|---|
| 0 | 0.24 ± 0.04[a] | – | – |
| 2 | 0.27 ± 0.11 | 0.32 ± 0.07 | 0.26 ± 0.07 |
| 4 | 0.35 ± 0.13 | 0.58 ± 0.09* | 0.74 ± 0.16* |
| 6 | 0.72 ± 0.14* | 0.75 ± 0.17* | 0.71 ± 0.06* |

[a]*mdr*1 and γ-actin RNA levels in the CEM/A7R cells exposed to 1.5 μg ml$^{-1}$ DOX, DAU and EPI for 2, 4 or 6 h were quantified using a Molecular Dynamics phosphorimager. The ratio of *mdr*1 to γ-actin is given as the mean ± s.d. (calculated from three Northern blotting analyses of the one experiment). These results were compared with the ratio of *mdr*1 to γ-actin in the untreated CEM/A7R cells (0.24 ± 0.04) and analysed using the Wilcoxon rank-sum test. *$P$<0.02 for marked samples. Similar results were obtained in three identical experiments.

**Figure 2** Effects of increasing doses of EPI on up-regulation of *mdr*1 gene expression in the CEM/A7R line. Cells in exponential growth phase were treated with (or without) EPI at doses ranging from 0.1 to 3.0 μg ml$^{-1}$ for 24 h. RNA was extracted and hybridised with $^{32}$P-labelled *mdr*1 and γ-actin as described previously.

**Figure 3** Effect of increasing doses of DAU or EPI on up-regulation of *mdr*1 in the CEM/A7 line. Cells in exponential growth phase were treated with (or without) 0.1–3.0 μg ml$^{-1}$ EPI (●–●) and DAU (▲–▲) for 24 h. RNA was extracted and analysed by Northern blotting as described in Materials and methods. The comparison of *mdr*1 expression was quantitated by scanning Northern blots on a phosphorimager as described previously. Each point represents the mean of triplicate slot blot analysis; bars give the standard deviation. Similar dose–response patterns were seen in a repeat experiment.

CEM/A7 cells (Figure 4). Exposed to 1 μg ml$^{-1}$ EPI or DAU, CEM/A7R cells showed a 3- to 4-fold increase in P-gp expression (mean channel fluorescence 177 and 144 respectively) compared with untreated CEM/A7R cells (mean channel fluorescence 45).

The stability of the increased levels of *mdr*1 and P-gp expression in response to DOX was tested 3 weeks after the CEM/A7R cells had been exposed to 1 μg ml$^{-1}$ DOX for 24 h. The treated cells were cultured in drug-free medium for 3 weeks before being subjected to a further analysis of *mdr*1

**Figure 4** Flow cytometric analysis of P-gp expression using MRK-16 binding (filled histogram) compared with an $IgG_{2a}$ control (unfilled histogram) in the CCRF-CEM, CEM/A7 and the CEM/A7R lines before and after exposure to EPI or DAU. P-gp expression was not seen in the CCRF-CEM parental cell line. CEM/A7R expressed much less P-gp (10-fold) than the CEM/A7 line. CEM/A7R cells were treated with (or without) 1.5 μg ml$^{-1}$ of EPI and DAU for 24 h before measurement of P-gp levels as described in Materials and methods. P-gp expression was increased more than 3-fold in the treated CEM/A7R cells compared with untreated controls.

**Figure 5** The stability of the increase in *mdr*1 and P-gp levels previously described was estimated by comparing the resistance profiles of treated and untreated cells 3 weeks after a 24 h exposure to 1.5 μg ml$^{-1}$ DOX. In the intervening 3 weeks both cell lines were maintained in drug-free medium. The percentage change in cell number of untreated (O – O) or treated cells (● – ●) exposed to increasing concentrations of EPI 3 weeks after a 24 h exposure to DOX relative to untreated controls is plotted. Points represent the means of triplicate determinations and bars the standard deviation.

expression and measurement of P-gp levels. The *mdr*1 mRNA and P-gp levels in the DOX-treated CEM/A7R cells remained 2- to 3-fold higher than the untreated CEM/A7R cells (data not shown), corresponding to the 3-fold increase in drug resistance demonstrated in a growth assay (Figure 5).

### Discussion

The human *mdr*1 gene can be induced by environmental stresses such as heat shock or arsenite (Chin *et al.*, 1990*b*; Kioka *et al.*, 1992*a*) and agents which affect cellular differentiation (Bates *et al.*, 1989; Mickley *et al.*, 1989). However, the effect of cytotoxic drugs on gene expression and in turn the MDR phenotype remain poorly understood. Two related hypotheses are equally plausible: selection of pre-existing MDR cells owing to the preferential growth advantage of low numbers of these cells in the presence of cytotoxic drugs or the induction of *mdr*1 expression in cells with low P-gp levels. While these two concepts are not mutually exclusive, the effect of cytotoxic drugs on the regulation of *mdr*1 gene is likely to be of critical importance in understanding how cytotoxic drugs should be used in this setting.

The studies reported herein were carried out in a variant MDR cell line (CEM/A7R) with very low levels of *mdr*1 gene expression and drug resistance (Table I, Figures 1 and 4). The line was originally derived from a classical MDR human leukaemia cell line (CEM/A7) initially selected for resistance to DOX by a stepwise selection procedure. The CEM/A7 line was maintained in conditioned medium containing 0.07 μg ml$^{-1}$ DOX (Zalcberg *et al.*, 1994). Induction of *mdr*1 mRNA could not be demonstrated in this cell line, presumably because of the continuous selection pressure. In addition, induction was not seen in the sensitive CCRF-CEM line (data not shown). A variant MDR cell line was developed by continuous culture of the CEM/A7 line in the absence of DOX. The subcloned variant cell line labelled CEM/A7R was stable in drug-free medium for over 12 months before conducting the experiments reported in this study. The CEM/A7R line expressed P-gp to a significantly lesser extent than the classical MDR line from which it was derived (Figures 1 and 4). This line appears to be a useful model for studying clinical drug resistance in so far as it does not have detectable amplification of the *mdr*1 gene and expresses very low levels of P-gp in the absence of a continuous selective pressure.

In order to assess the effects of cytotoxic drugs on *mdr*1 expression, the ratio of *mdr*1 RNA to an internal RNA (actin) control was quantitatively analysed following the exposure of cells to various drugs by scanning Northern blots on a phosphorimager. A significant increase (2-fold) in *mdr*1 expression was observed within 4 h of exposure of CEM/A7R cells to 1.5 μg ml$^{-1}$ DAU or EPI and 6 h to 1.5 μg ml$^{-1}$ DOX (Table II). A 3- to 4-fold increase in *mdr*1 expression was observed after an 8 h exposure to 1.5 μg ml$^{-1}$ DOX, DAU or EPI (Figure 1a and b). Increases in *mdr*1 levels also resulted in an increase in P-gp expression (Figure 4). Neither VLB nor VIN had any effect on *mdr*1 expression after exposure to these drugs for 8 h (Figure 1a and b). In view of the cell doubling time of 24 h for the CEM/A7R line, these changes in *mdr* expression are occurring too quickly to be explained by cell selection, suggesting that the induction of drug resistance may be a first step in the development of clinical resistance, with selection occurring as a secondary event.

Recently, Chin *et al.* (1990*a*) reported the induction of *mdr*1 expression in a rodent cell line as early as 2 h after exposure to anthracyclines. They observed levels as high as 30- to 100-fold greater than in untreated controls, although no detectable increase in *mdr*1 expression was seen in human tumour cells. They concluded that species differences in the promoter sequence and/or consensus binding sites (Ueda *et al.*, 1987; Hsu *et al.*, 1989; Cornwell, 1990; Ikeguchi *et al.*, 1991) may account for the induction of *mdr*1 expression in rodent but not human cell lines. Alternatively, induction may have been demonstrable in our model because of the inherent capacity of T cells to alter gene expression in response to environmental stimuli.

In the study reported by Chin *et al.* (1990*a*), no change in expression of the *mdr*1 gene was seen following exposure of rodent cells to vinca alkaloids or epipodophyllotoxins (Chin *et al.*, 1990*a*). We also failed to observe any increase in *mdr*1 expression in response to VIN or VLB after an 8 h exposure, although VP16 was associated with an increase in *mdr*1 expression in our system. It is possible that these findings relate to the sensitivity of the assay system used and hence further clarification of this phenomenon will depend on more detailed dose and time course experiments now under way in our laboratory.

The precise mechanism underlying up-regulation of the *mdr*1 gene by anti-cancer drugs in these human cells is unclear but may operate at either the transcriptional or post-transcriptional level. The increse in *mdr*1 levels after the exposure of CEM/A7R cells to EPI for 4 h suggests that changes in the rate of transcription may be important in this model. The possible mechanisms responsible for the rapid induction of *mdr*1 expression are currently under investigation in our laboratory. In addition, as variable responses to anthracycline analogues, all of which represent substrates for P-gp, have not previously been identified, studies to clarify the interaction between these analogues and the *mdr*1 promoter are also under way.

**Acknowledgements**

This study was supported in part by the Anti-Cancer Council of Victoria and Department of Veterans Affairs, Canberra.

### References

BATES SE, MICKLEY LA, CHEN YN, RICHERT N, RUDICK J, BIEDLER JL AND FOJO AT. (1989). Expression of a drug resistance gene in a human neuroblastoma cell line: modulation by retinoic acid-induced differentiation. *Mol. Cell. Biol.*, **9**, 4337-4344.

CHAN SLH, THORNER PS, HADDAD G AND LING V. (1990). Immunohistochemical detection of P-glycoprotein: prognostic correlation in soft tissue sarcoma of childhood. *J. Clin. Oncol.*, **8**, 689-704.

CHAUDHARY PM AND RONINSON IB. (1993). Induction of multidrug resistance in human cells by transient exposure to different chemotherapeutic drugs. *J. Natl Cancer Inst.*, **85**, 632-639.

CHEVILARD S, VIELH P, BASTIAN G AND COPPEY J. (1992). A single 24h contact time with adriamycin provokes the emergence of resistant cells expressing the Gp 170 protein. *Anticancer Res.*, **12**, 485-500.

CHIN KV, CHAUHAN SS, PASTAN I AND GOTTESMAN MM. (1990*a*). Regulation of mdr RNA levels in response to cytotoxic drugs in rodent cells. *Cell Growth Different.*, **1**, 361-365.

CHIN KV, TANAKA S, DARLINGTON G, PASTAN I AND GOTTESMAN MM. (1990*b*). Heat shock and arsenite increase expression of the multidrug resistance (MDR1) gene in human renal carcinoma cells. *J. Biol. Chem.*, **265**, 221-226.

CHOMCZYNSKI P AND SACCHI N. (1987). Single-step method of RNA isolation by acid guanidinium thiocyanate-phenol-chloroform extraction. *Anal. Biochem.*, **162**, 156-159.

CORNWELL M. (1990). The human multidrug resistance gene: sequences upstream and downstream of the initiation site influence transcription. *Cell Growth Different.*, **1**, 607-615.

DEUCHARS KL AND LING V. (1989). P-glycoprotein and multidrug resistance in cancer chemotherapy. *Semin. Oncol.*, **16**, 156-165.

FERRANDIS E AND BENARD J. (1993). Activation of the human MDR1 gene promoter in differentiated neuroblasts. *Int. J. Cancer*, **54**, 987-991.

FOLEY GE, LAZARUS H, FARBER S, UZMAN BG, BOONE BA AND MCCARTHY RE. (1965). Continuous culture of human lymphoblasts from the peripheral blood of a child with acute leukemia. *Cancer*, **18**, 522-529.

FORD JM AND HAIT WN. (1990). Pharmacology of drugs that alter multidrug resistance in cancer. *Pharmacol. Rev.*, **42**, 156-199.

GOASGUEN JE, DOSSOT J-M, FARDEL O, LE MEE F, LE GALL E, LEBLAY R, LEPRISE PY, CHAPERON J AND FAUCHET R. (1993). Expression of the multidrug resistance-associated P-glycoprotein (P-170) in 59 cases of *de novo* acute lymphoblastic leukemia; prognostic implications. *Blood*, **81**, 2394-2398.

GOLDSTEIN LJ, PASTAN P AND GOTTESMAN MM. (1992). Review article: multidrug resistance in human cancer. *Crit. Rev. Oncol. Haematology*, **12**, 243-253.

HAYES JD AND WOLF CR. (1990). Molecular mechanisms of drug resistance. *Biochem. J.*, **272**, 281-295.

HSU SI-H, LOTHSTEIN L AND HORWITZ SB. (1989). Differential overexpression of three mdr gene family members in multidrug-resistant J774.2 mouse cells. Evidence that distinct P-glycoprotein precursors are encoded by unique mdr genes. *J. Biol. Chem.*, **264**, 12053-12062.

HU XF, MARTIN TJ, BELL DR, DE LUISE M AND ZALCBERG JR. (1990). Combined use of cyclosporin A and verapamil in modulating multidrug resistance in human leukemia cell lines. *Cancer Res.*, **50**, 2953-2957.

IKEGUCHI M, TEETER M, ECKERSBERG T, GANAPATATHI R AND KUO MT. (1991). Structural and functional analyses of the promoter of the murine multidrug resistance gene mdr3/mdr1a reveal a negative element containing the AP-1 binding site. *DNA Cell Biol.*, **10**, 639-649.

KIOKA N, HOSOKAWA N, KOMANO T, HIRAYOSHI K, NAGATA K AND UEDA K. (1992*a*). Quercetin, a bioflavonoid, inhibits the increase of human multidrug resistance gene (MDR1) expression caused by arsenite. *FEBS Lett.*, **3**, 307-309.

KIOKA N, YAMANO Y, KOMANO T AND UEDA K. (1992*b*). Heat-shock responsive elements in the induction of multidrug resistance gene (*MDR* 1). *FEBS*, **301**, 37-40.

KOHNO K, SATO S, TAKANO H, MATSUO K-I AND KUWANO M. (1989). The direct activation of human multidrug resistance gene (MDR1) by anticancer agents. *Biochem. Biophys. Res. Commun.*, **165**, 1415-1421.

LICHT T, CIEBIG HH, BROSS K, HERRMANN F, BERGER DP, SHOEMAKER R AND SANN M. (1991). Induction of multidrug resistance during anti-neoplastic chemotherapy in vitro. *Int. J. Cancer*, **49**, 630-637.

MICKLEY LA, BATES SE, RICHERT ND, CURRIER S, TANAKA S, FOS F, ROSEN N AND FOJO AT. (1989). Modulation of the expression of a multidrug resistance gene (mdr-1/P-glycoprotein) by differentiating agents. *J. Biol. Chem.*, **264**, 18031-18040.

MIYAZAKI M, KOHNO K, UCHIUMI T, TANIMURA H, MATSUO K, NASU M AND KUWANO M. (1992). Activation of the human multidrug resistance -1 gene promoter in response to heat shock stress. *Biochem. Biophys. Res. Commun.*, **187**, 677-684.

SLATER LM, SWEET P, STUPECKY M, WELZEL MW AND GUPTA S. (1986). Cyclosporin A corrects daunorubicin resistance in Ehrlich ascites carcinoma. *Br. J. Cancer*, **54**, 235-238.

TANIMURA H, KOHNO K, SATO S-i, UCHIUMI T, MIYAZAKI M, KOBAYASHI M AND KUWANO M. (1992). The human multidrug resistance I promoter has an element that responds to serum starvation. *Biochem. Biophys. Res. Comm.*, **183**, 917-924.

TSURUO T, IIDA H, TSUKAGOSHI S AND SAKURAI Y. (1981). Overcoming of vincristine resistance in P388 leukemia *in vivo* and *in vitro* through enhanced cytotoxicity of vincristine and vinblastine by verapamil. *Cancer Res.*, **41**, 1967-1972.

UEDA K, PASTAN I AND GOTTESMAN M. (1987). Isolation and sequence of the promoter region of the human multidrug-resistance (P-glycoprotein) gene. *J. Biol. Chem.*, **262**, 17432-17436.

ZALCBERG JR, HU XF, WALL DM, MIRSKI S, COLE S, NADALIN G, DE LUISE M, PARKIN JD, VRAZAS V, CAMPBELL L AND KANTHARIDIS P. (1994). Cellular and karyotypic characterization of two doxorubicin resistant cell lines isolated from the same parental human leukemia cell line. *Int. J. Cancer*, **57**, 522-528.